# Protective effect of *Launaea procumbens* (L.) on lungs against CCl_4_-induced pulmonary damages in rat

**DOI:** 10.1186/1472-6882-12-133

**Published:** 2012-08-21

**Authors:** Rahmat Ali Khan

**Affiliations:** 1 Department of Biotechnology, Faculty of Biological Sciences, University of Science and Technology Bannu, Bannu, KPK, Pakistan

**Keywords:** Launaea procumbens, Lungs, CCl_4_, Antioxidant enzymes, TBARS, GSH

## Abstract

**Background:**

*Launaea procumbens* (L.) is traditionally used in the treatment of various human ailments including pulmonary damages. The present study was arranged to evaluate the role of *Launaea procumbens* methanol extract (LME) against carbon tetrachloride (CCl_4_) induced oxidative pulmonary damages in rat.

**Methods:**

36 Sprague–Dawley male rats (170-180 g) were randomly divided into 06 groups. After a week of acclamization, group I was remained untreated while group II was given olive oil intraperitoneally (i.p.) and dimethyl sulfoxide **(**DMSO) orally, groups III, IV, V and VI were administered CCl_4_, 3 ml/kg body weight (30% in olive oil i.p.). Groups IV, V were treated with 100 mg/kg, 200 mg/kg of LME whereas group VI was administered with 50 mg/kg body weight of rutin (RT) after 48 h of CCl_4_ treatment for four weeks. Antioxidant profile in lungs were evaluated by estimating the activities of antioxidant enzymes; catalase (CAT), peroxidase (POD), superoxide dismutase (SOD), glutathione-S-transferase (GST), glutathione reductase (GSR), glutathione peroxidase (GSH-Px), quinone reductase (QR) and reduced glutathione (GSH). CCl_4_-induced lipid peroxidation was determined by measuring the level of thiobarbituric acid reactive substances (TBARS) with conjugation of deoxyribonucleic acid (DNA) damages, argyrophilic nucleolar organizer regions (AgNORs) counts and histopathology.

**Results:**

Administration of CCl_4_ for 6 weeks significantly *(p < 0.01)* reduced the activities of antioxidant enzymes and GSH concentration while increased TBARS contents and DNA damages in lung samples. Co-treatment of LME and rutin restored the activities of antioxidant enzymes and GSH contents. Changes in TBARS concentration and DNA fragmentation were significantly *(p < 0.01)* decreased with the treatment of LME and rutin in lung. Changes induced with CCl_4_ in histopathology of lungs were significantly reduced with co-treatment of LME and rutin.

**Conclusion:**

Results of present study revealed that LME could protect the lung tissues against CCl_4_-induced oxidative stress possibly by improving the antioxidant defence system.

## Background

Reactive oxygen species (ROS) and reactive nitrogen species (RNS) are produced as byproducts during normal metabolism, exposure of sunlight, ultraviolet light, ionizing radiation and toxic chemicals [1]; probably causes chronic diseases and pulmonary damages [2]. Various reports revealed that pneumotoxic substances such as carbon tetrachloride [3], paraquat [4] and bleomycin [5] play a critical role in pulmonary injury. Carbon tetrachloride (CCl_4_) is a potent environmental hepatotoxin, used as industrial organic solvents [6] cause liver, kidney and lungs dysfunction in workers [7]. It has been established that trichloromethyl (CCl_3_) radical and chloride (Cl) are formed as a result of the metabolic conversion of CCl_4_ by cytochrome P-450 [8]. These free radicals react with polyunsaturated fatty acids (PUFA) of lung membranes and enhance lipid peroxidation (TBARS), DNA fragmentation [9], deplete activities of antioxidant enzymes such as CAT, SOD, GSH-Px, GSR and amount of tissue soluble proteins [10]. Medicinal plants and polyphenolic compounds isolated from medicinal plants play important role in the treatment of various human ailments and in the detoxification of the products (the intermediate and final) of oxidative stress [11] as well as ameliorate lungs oxidative damages in experimental rats [3,12].

*Launaea procumbens* (LP) is one of important medicinal plant widely spread in waste places, vacant lots and in cultivated fields through out Pakistan. Ayurvedic and herbal medicine prepared from this plant promote self healing, good health and longevity, as well as used as a food ingredient [13]. It has been used in the treatment of nephritis, pulmonary fibrosis, hormonal balance and sexual diseases by local healer in Pakistan [14]. Phytochemistry of LP revealed the presence of salicylic acid, vanillic acid, 2-methyl-resercinol and gallic acid [15]. These compounds have spasmogenic, cardiovascular, anti-carcinogenic, anti-inflammatory, and antioxidant properties to scavenge reactive oxygen species. The present study was therefore arranged to investigate the protective effects of LP on lungs against CCl_4_-induced oxidative damage in rats.

## Methods

### LP collection and extraction

Aerial parts of LP were collected during June 2010, identified and a specimen was submitted at Herbarium of Pakistan, Quaid-i-Azam University (QAU) Islamabad, Pakistan. Leaves were shade dried at room temperature and ground mechanically. 2 kg of the powder was extracted twice in 5 liter of methanol with random shaking for a week and evaporated through rotary evaporator after filtration by Whatmann filters paper No. 45; to get crude methanolic extract (LME). LME is stored at 4°C for *in vivo* studies.

### Animals and experimental design

36 Sprague–Dawley male rats (170-1800 g) were purchased from NIH, Islamabad, Pakistan and brought to animal house of Quaid-i-Azam University Islamabad. After one week of acclamization under standard laboratory conditions (12 h light/darkness; at 25 ± 3°C), with free access of diet and water, they were randomly divided into 06 groups according to study protocol as approved by ethical committee of Quaid-i-Azam University, Islamabad. Group I remained untreated (control) while group II was given olive oil intraperitoneally and DMSO orally, groups III-VI were administered CCl_4_, 3 ml/kg body weight (30% in olive oil i.p.). Groups IV and V were treated with 100 mg/kg and 200 mg/kg of LME while group VI was treated with 50 mg/kg body weight of RT after 48 h of CCl_4_ treatment. These treatments were carried out twice a week for four weeks. After 24 h of the last treatment, all the animals were weighted, sacrificed; their lungs were removed, weighted and perfused in ice-cold saline solution. Half of lung tissue was treated with liquid nitrogen for further enzymatic and DNA damage analysis while the other portion was processed for histology.

### Assessment of antioxidant status

Tissue of lungs was homogenized in phosphate buffer (pH 7.4) and centrifuged at 12,000 × g at 4°C for 30 min to get tissue homogenate. Tissue soluble protein concentration of lung homogenate was obtained [16] while antioxidant status was determined by estimation of antioxidant activities of CAT and POD [17], SOD [18], GST [19], GSR [20], GSH-Px [21], H_2_O_2_ concentration [22] and activity of QR [23]. Oxidative status was determined using estimation of GSH [24] while lipid peroxidation (TBARS) was [25] in lung homogenates.

### Nitrite assay

Griess reagent was used for determination of nitrite contents. The reagent includes 0.3 M NaOH and 5% ZnSO_4_ used as griess reagent. Concentration of nitrite contents was expressed using sodium nitrite standard curve.

### DNA ladder assay

Protocol of Wu et al. [26] was used for isolation of DNA to determine DNA damages. 5 μg DNA extracted from each sample of different groups separately loaded in 1.5% agarose gel for 45 min and photographed, using digital camera under gel doc system.

### % Quantification of DNA fragmentation

Quantification of % DNA fragmentation was carried out using tris triton EDTA (TTE), trichloro acetic acid (TCA) and diphenylamine (DPA) as regents. OD of DNA was checked with a spectrophotometer (Smart spec ^TM^ Plus, catalog # 170–2525) at 600 nm [26].

### AgNORs count

Silver staining technique was used according to [27]. During NORs staining, unstained fixed slides were dewaxed with xylene and hydrated in decrease ethanol concentration (90, 70 and 50%) and washed. After drying slides were treated with one drop of colloidal solution (2% gelatin and 1% formic acid) and two drops of 50% AgNO_3_ solution onto the slide and incubated at 35°C for 8–12 min. The progressive staining was followed under light microscope to get golden colored nuclei and brown/black NORs at 100 × magnification and counted number of NORs per cell.

### Morphological study of lungs

Microscopic studies of lung tissues were carried out by the protocol as used by Khan et al. [3] with some modifications.

### Statistical analysis

Data were expressed as mean and standard error (SE) and ANOVA test were used to analyze the difference among various treatments, with least significance difference (LSD) at 0.05 and 0.01 as a level of significance. SPSS ver. 14.0 (Chicago, IL, USA) and Microsoft Excel 2007 (Roselle, IL, USA) were used for the statistical and graphical evaluations.

## Results

### Effect of LME on lungs protein and antioxidant enzymes in rat

**Figure 1:**
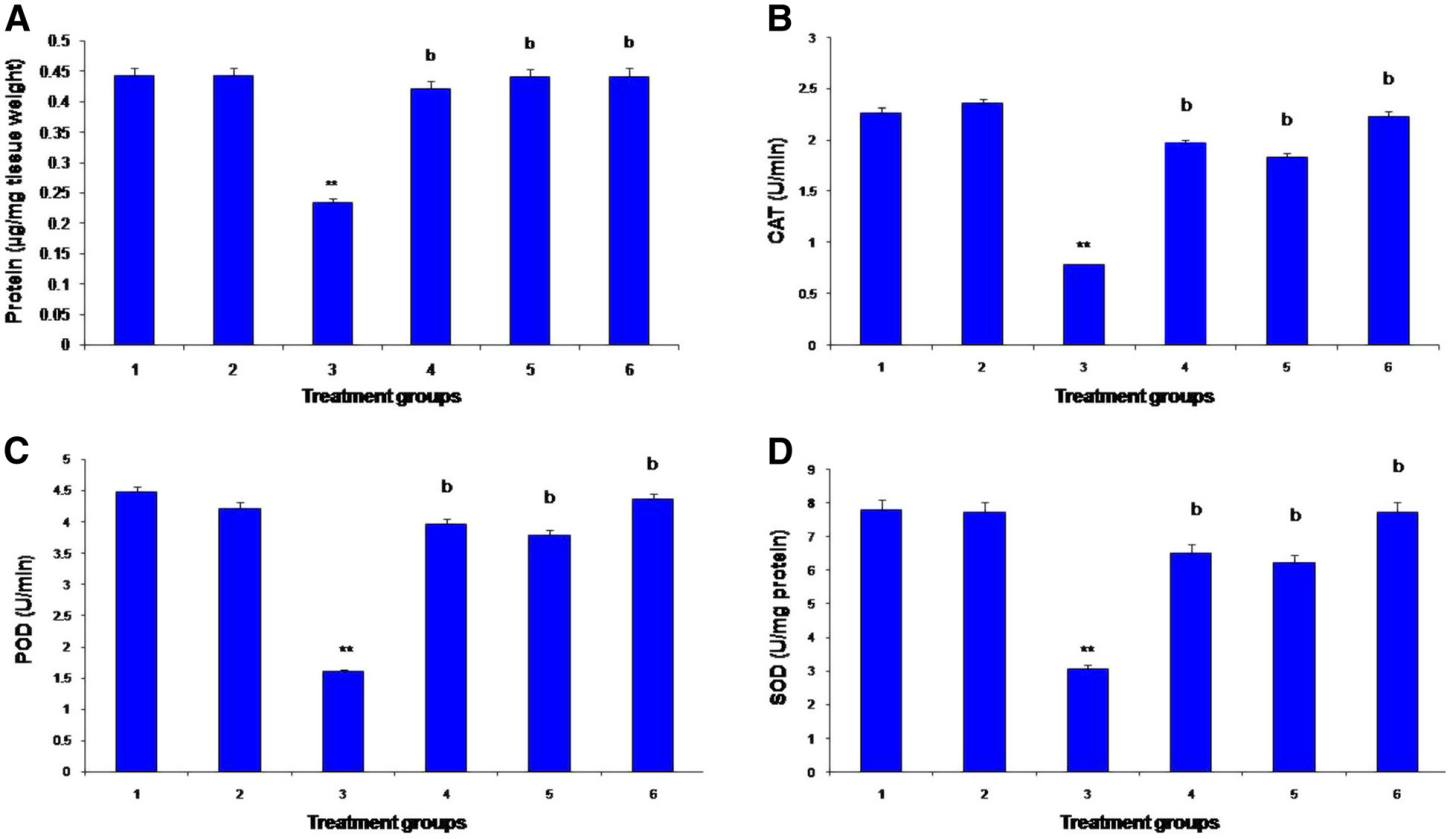
**(A-D) Effect of LME treatments on tissue protein (A), antioxidant enzymes; catalase (CAT) (B), peroxidase (POD) (C) superoxide dismutase (SOD) (D) in lungs of various groups in rat.** I: control; II: vehicle (olive oil + DMSO); III: CCl_4_ 3 ml/kg; IV: CCl_4_ + rutin 50 mg/kg; V: CCl_4_ + LME 100 mg/kg; VI: CCl_4_ + LME 200 mg/kg; ** *(P < 0.01)* show significance from the control group. *b (P < 0.01)* show significance from the CCl_4_ group. Mean ± SE (n = 06 rats).

Figure 1 shows changes in lung protein and activities of antioxidant enzymes in all the experimental groups of rat. Administration of CCl_4_ significantly *(p < 0.01)* decreased the tissue soluble protein, activities of CAT, POD and SOD as compared to normal rats. Treatment with LME markedly augmented *(p < 0.01)* the effects of CCl_4_ intoxicity, and restored the amount of tissue soluble protein, activities of CAT, POD and SOD in lung tissues in a dose dependent way. Treatment of RT also showed significant protection *(p < 0.01)* in the improvement of tissue protein and activities of antioxidant enzymes comparatively to control group.

### Effect of LME on lungs GSH-Px, GST, QR and GSR

Changes in the activities of different enzymes viz; GST, GSR, GSH-Px and QR are shown in Table 1. CCl_4_ treatment to rats considerably *(p < 0.01)* depleted the activities of GSR, GST, GSH-Px and QR. Treatment of rats with different doses of LME alleviated the toxic effects of CCl_4_ by increasing the activity of GSH-Px, GSR, QR and GST as compared to the CCl_4_ group. Similar observations were deliberated in rats having a dose (50 mg/kg b.w.) of RT used in this experiment.

**Table 1:** Effect of LME on lungs GSH-Px, GSR, GST and QR

**Treatment**	**GSH-Px (nM/mg protein)**	**GSR (nM/min/mg protein)**	**GST (nM/min/mg protein)**	**QR (nM/min/mg protein)**
Control	103.17 ± 0.60++	65.83 ± 1.33++	54.83 ± 10.1++	81.00 ± 1.65++
Olive oil + DMSO	99.000 ± 0.577++	64.17 ± 1.33++	52.667 ± 8.2++	80.00 ± 1.71++
3 ml/kg CCl_4_	54.500 ± 0.764**	29.3 ± 0.494**	35.500 ± 7.4**	39.33 ± 1.73**
100 mg/kg LME + CCl_4_	77.500 ± 0.764++	54.3 ± 0.88++	45.333 ± 7.1++	67.5 ± 1.9++
200 mg/kg LME + CCl_4_	98.567 ± 0.464++	62.1 ± 1.3++	51.42 ± 11.4++	77.58 ± 1.71++
50 mg/kg RT + CCl_4_	96.333 ± 0.882++	62.17 ± 1.62++	51.33 ± 13.3++	77.33 ± 1.89++

Mean ± SE (n = 6 number).

** indicate significance from the control group at *p <* 0.01 probability level.

++ indicate significance from the CCl_4_ group at *p <* 0.01 probability level.

### Effect of LME on lung GSH, TBARS, H_2_O_2_ and nitrite contents in rat

Changes in the content of GSH, TBARS, H_2_O_2_ and nitrite in lungs of rat are shown in Table 2. Administration of CCl_4_ significantly depleted *(p < 0.01)* the GSH while increased markedly *(p < 0.01)* the tissue nitrite, TBARS and H_2_O_2_ contents as compared to normal rats. Administration of LME and RT in CCl_4_ treated rats considerably (p < 0.01) augmented the toxic effects of CCl_4_, restored the GSH, TBARS, nitrite and H_2_O_2_ contents towards the non treated control group.

**Table 2:** **Effect of LME on lungs TBARS, H**_**2**_**O**_**2**_**, GSH and nitrite contents**

**Treatment**	**TBARS (nM/min/mg protein)**	**H**_**2**_**O**_**2**_**(nM/min/mg tissue)**	**GSH (μM/g tissue)**	**Nitrite (μM/ml)**
Control	19.51 ± 0.17++	5.71 ± 0.41++	0.53 ± 0.014++	30.36 ± 0.99++
Olive oil + DMSO	19.83 ± 0.30++	5.32 ± 0.71++	0.52 ± 0.010++	31.8 ± 1.23++
3 ml/kg CCl_4_	39.16 ± 0.70**	14.11 ± 0.31**	0.25 ± 0.004**	59.4 ± 1.37**
100 mg/kg LME + CCl_4_	24.25 ± 0.62++	6.21 ± 0.61++	0.53 ± 0.009++	33.5 ± 0.69++
200 mg/kg LME + CCl_4_	20.50 ± 0.42++	6.52 ± 0.41++	0.52 ± 0.007++	34.0 ± 1.11++
50 mg/kg RT + CCl_4_	25.66 ± 0.33++	5.92 ± 0.31++	0.50 ± 0.01++	35.2 ± 1.38++

Mean ± SE (n = 6 number).

** indicate significance from the control group at *p <* 0.01 probability level.

++ indicate significance from the CCl_4_ group at *p <* 0.01 probability level.

### Effect of LME on body weight, lung weight, relative lung weight, AgNORs count and % DNA fragmentation in rat

Toxic effects of CCl_4_ administration in rat on % changes in body weight, lung weight, relative lung weight, AgNORs count and % DNA fragmentation are presented in Table 3. Administration of CCl_4_ significantly increased *(p < 0.01)* lung weight, relative lung weight, AgNORs count and %DNA fragmentation while decreased the % body weight of rats as compare to normal rats. Post-treatment of LME at 100 mg/kg b.w and 200 mg/kg b.w, dose improved the CCl_4_ intoxication and extensively reduced *(p < 0.01)* the lung weight, relative lung weight, % DNA damages and AgNORs count as compare to CCl_4_ group.

**Table 3:** Effect of LME on lung weight, relative lung weight, AgNORs count and DNA damages in rat

**Treatment**	**% increase in Body weight**	**Lung weight (g)**	**Relative lung weight**	**DNA Fragmentation%**	**AgNORs (NORs/cell)**
Control	26.0 ± 0.80++	2.56 ± 0.006++	0.026 ± 0.00006++	7.07 ± 0.43++	1.26 ± 0.15++
Olive oil + DMSO	25.9 ± 0.63++	2.55 ± 0.008++	0.025 ± 0.00008++	6.93 ± 1.42++	1.19 ± 0.07++
3 ml/kg CCl_4_	18.6 ± 0.72**	3.89 ± 0.020**	0.038 ± 0.00020**	29.80 ± 1.41**	7.8 ± 0.52**
100 mg/kg LME + CCl_4_	22.8 ± 0.81++	2.91 ± 0.025++	0.029 ± 0.00025++	18.07 ± 1.33++	3.5 ± 0.13++
200 mg/kg LME + CCl_4_	25.6 ± 0.73++	2.56 ± 0.007++	0.026 ± 0.00007++	7.40 ± 1.56++	2.95 ± 0.06++
50 mg/kg RT + CCl_4_	24.5 ± 0.46++	2.09 ± 0.020++	0.020 ± 0.00020++	7.10 ± 1.50++	2.03 ± 0.09++

Mean ± SE (n = 6 number).

** indicate significance from the control group at *p < 0.01* probability level.

++ indicate significance from the CCl_4_ group at *p < 0.01* probability level.

### Effect of LME on lung DNA damages in rat

**Figure 2:**
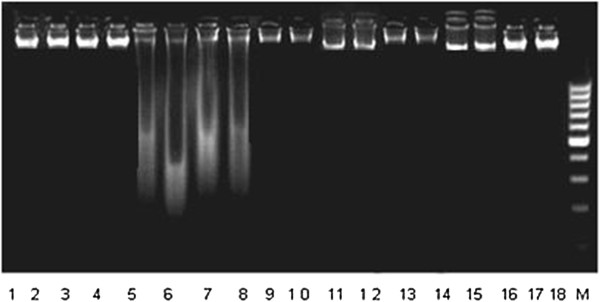
**Agarose gel showing DNA damage by CCl**_**4 **_**and preventive effect of *Launaea procumbens *extracts in different groups.** Lanes (from left), Control (1–4), CCl_4_ (5–8), 100 mg/kg LME (9, 12) 200 mg/kg LME (13, 16), 50 mg/kg RT (17, 18), DNA marker (M).

DNA damages in the lung tissues of rats in different experimental groups are shown in Figure 2. DNA ladder assay in control group (Lane 1–4) show no changes while extensive DNA damages were found in CCl_4_ group as depicted by (Lane 5,6). Post-administration of 100 mg/kg, 200 mg/kg b.w, LME and 50 mg/kg b.w, rutin reduced the DNA damages, dose dependently, as shown by DNA band pattern of different groups comparatively to CCl_4_ group (Lane 7–10), (Lane 11–14) and (Lane 15–18) respectively.

### Effect of LME on lung morphology

**Figure 3:**
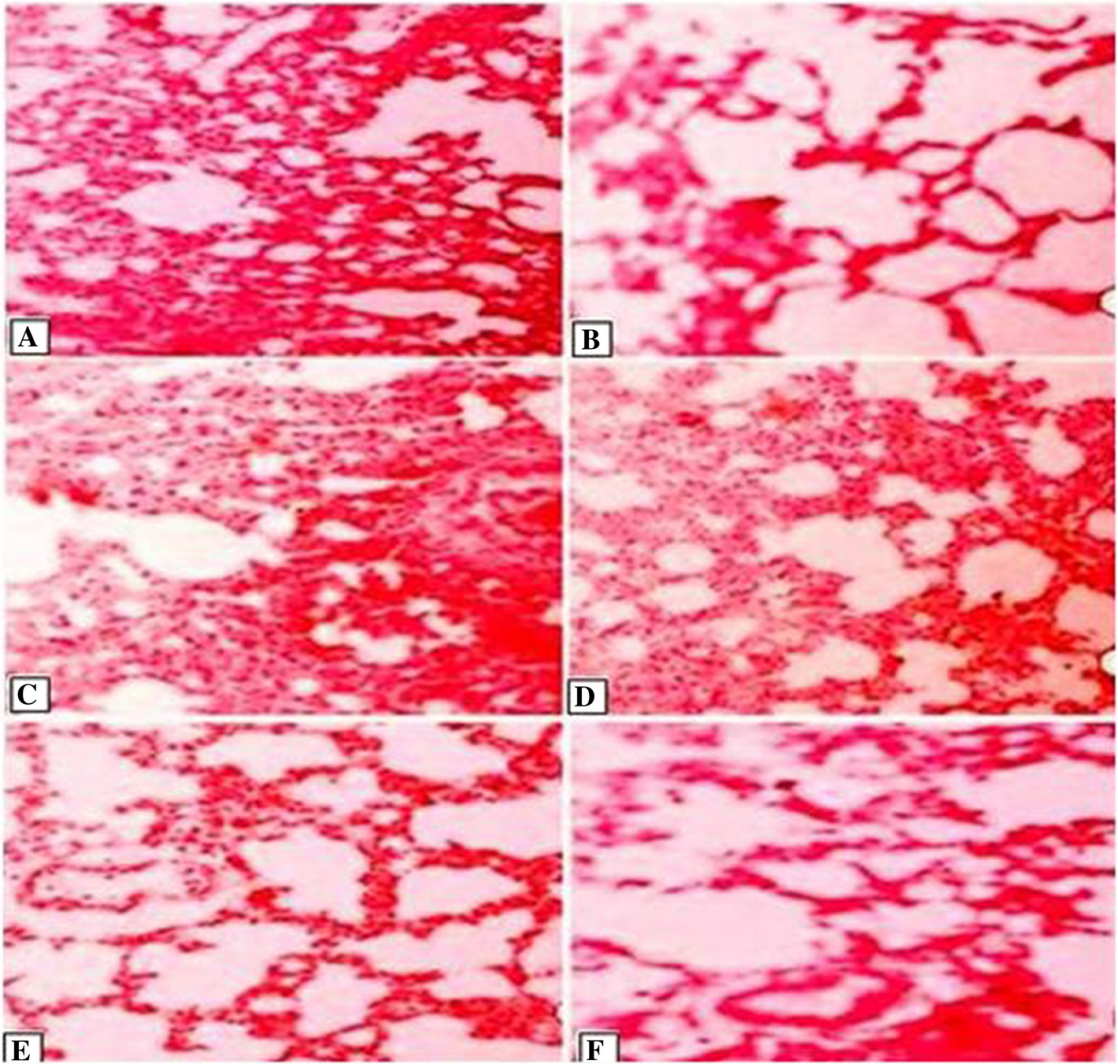
**Effect of LME on lung morphology; Slides (from left) Control (A), CCl**_**4 **_**(B, C), 100 mg/kg LME (D) 200 mg/kg LME (E) 50 mg/kg RT (F).**

The thin sections of control having normal alveoli with thin intralveolar septum, type I and type II pneumocytes were also clearly observed (Figure 3A). The alveolar macrophages were also prominent and the alveolar bronchioles show their normal shape with inner epithelium. Treatment of CCl_4_ in the lungs induced the degeneration of the alveolar septa, disruption of the connective tissues, elastic fibers and the congestion of the blood capillaries which were also blocked with large aggregation of the blood cells (Figure 3B & C). Administrations of 100 mg/kg, 200 mg/kg LME and 50 mg/kg b.w. RT reduced the toxic effects of CCl_4_ and reduced injuries were observed in the lung tissues of these groups. The ameliorating effects of the fractions were more pronounced at the higher dose of LME (Figure 3D, E & F). Most areas of the lungs showed the normal alveolar spaces, alveolar and bronchioles with minor cell degeneration, PNI, PNII but a less marked thickening was still observed in the intralveolar septum (Table 4).

**Table 4:** Effect of LME on lung morphology

**Treatment**	**A b**	**DCT**	**DEF**	**CBC**	**ABC**	**PE**	**PF**
Control	-	-	-	-	-	-	-
Olive oil + DMSO	-	-	-	-	-	-	-
3 ml/kg CCl_4_	+++	+++	+++	+++	+++	+++	++
100 mg/kg LME + CCl_4_	−/+	-	-	−/+	-	-	-
200 mg/kg LME + CCl_4_	-	-	-	-	-	−/+	-
50 mg/kg RT + CCl_4_	−/+	-	-	-	-	-	-

**-,** normal; +/−, mild; +++, severe disruption.

AB (alveolar breakage), DCT (degeneration of connective tissue), DEF (damages of elastic fiber), CBC (congestion of blood capillaries), ABC (aggregation of blood cells), DIEAB (disorganized inner epithelium of alveolar bronchiole, PE (pulmonary edema), PF (pulmonary fibrosis.

## Discussion

Metabolism is a necessary process of living organisms for energy production; however normal metabolisms produce various reactive oxygen species (ROS) such as superoxide radicals (O_2_·-), hydrogen peroxide (H_2_O_2_) and hydroxyl radicals (OH·). In small amounts, these ROS are beneficial in signal transduction and growth regulation. However, large amount of ROS produced oxidative stress, attack various biomolecules [28]. ROS produced from CCl_4_ like trichloromethyl radical (·CCl_3_) and peroxy trichloromethyl radical (·OOCCl_3_) cause oxidative damages in lungs rats probably disturbing antioxidant status [3]. Antioxidant enzymes such as CAT, POD and SOD play key role in detoxification and protect lungs tissue from oxidative damages [29]. Data of the present study revealed that administration of CCl_4_ depleted activity of antioxidant enzymes; CAT, POD and SOD. Co-administration of various concentration of LME ameliorated the activities of antioxidant enzymes dose dependently, might be due to the presence of phenolic and polyphenolic compounds. Various other studies revealed similar reports [30,31]. Toxic metabolites of drugs and xenobiotic are metabolized by glutathione system (reduced glutathione, glutathione reductase, glutathione peroxidase and glutathione-S-transferase). In the present study we get marked decreased in activities of GST, GSR, GSH-Px and QR. Administration LME ameliorated the CCl_4_ toxicity, thereby increases the activity of GST, GSR, GSH-Px and QR. Adewole et al. [8] reported similar observations during administration of melatonin against CCl_4_ induced oxidative stress. Free radicals cause lipid peroxidation, elevate TBARS and deplete tissue GSH contents [32]. In the present study contents of GSH were considerably depleted while amplified the TBARS and H_2_O_2_ contents by induction of CCl_4_ comparatively to the control group in this study. Administration of different concentrations of LME extensively increased the GSH contents and decreased the TBARS and H_2_O_2_ contents. Similar observations were reported during co-treatment of plant extracts against CCl_4_ induced damages in rats [33]. Lipid peroxidation induced by CCl_4_ not only disturbs protein but diffuse into nucleic acid causes DNA fragmentation [34,35] which might lead to pulmonary damages. In the present study DNA fragmentation induced by CCl_4_ are ameliorated, with LME significantly as was reported by Khan et al. [9]. Quantification of AgNORs proteins per cell has been used in the diagnosis of oxidative lesions [36,37]. In this study co-administration of LME significantly augmented NORs/cell as was altered with treatment of CCl_4_ in lungs. Khan et al. [9] reported similar results during administration *Digera muricata* in rats.

Extensive variations were observed during histopathalogical study of rat lungs. Treatment of CCl_4_ caused destruction of alveolar septa and congestion of blood capillaries. As a result blood cells and collagen fibers are accumulated at various places leads to endemic condition. Similar observation was found in other study during CCl_4_ administraion in rat lungs [3,38]. Co-treatment with LME repaired the pulmonary damages; showing normal spaces in alveoli, reduced cellular degeneration of alveoli and bronchioles as well as normalized pneumocytes as were reported by Khan et al. [3] during *Sonchus asper* administration against CCl_4_-induced injuries in rats.

## Conclusion

Our results propose that LME comprised of bioactive compounds; presenting protective effects against CCl_4_ induced toxic affects in lungs of rat. Further studies of isolation and purification of these constituents are in progress in our lab.

## Competing interest

The authors declare that they have no competing interests.

## Authors’ contributions

RAK made significant contribution to acquisition of data, analysis, conception, design and drafting of the manuscript. The authors read and approved the final manuscript.

## Pre-publication history

The pre-publication history for this paper can be accessed here:

http://www.biomedcentral.com/1472-6882/12/133/prepub
